# Oral rehydration salt use and its correlates in low-level care of diarrhea among children under 36 months old in rural Western China

**DOI:** 10.1186/1471-2458-13-238

**Published:** 2013-03-19

**Authors:** Wenlong Gao, Hong Yan, Duolao Wang, Shaonong Dang

**Affiliations:** 1 Department of Epidemiology and Health Statistics, School of Public Health, College of Medicine, Xi’an Jiaotong University, PO Box 46, Xi’an, Shaanxi 710061, PR China; 2 Department of Medical Statistics, London School of Hygiene and Tropical Medicine, London, WC1E 7HT, UK

## Abstract

**Background:**

Since 2000, there has been a decline in the proportion of oral rehydration salts (ORS) therapy in childhood diarrhea. How to sustain and achieve a high level of ORS therapy continues to be a challenge.

**Methods:**

The data of 14112 households and 894 villages in 45 counties across 10 provinces of Western China were collected in 2005. Generalized estimated equation logistic regression models were used to identify the determinants of ORS use in home-based and village-level care.

**Results:**

The therapy rate of ORS was 34.62%. This rate in home-based care (HBC) was significantly lower than that in village-level care (VLC), township-level care or county-level-or-above care. The children in the families with several pre-school-aged children (OR = 0.29 95% CI: 0.10, 0.86) or of the smaller age (12 vs 36 months: OR = 0.10 95% CI 0.02, 0.41; 24 vs 36 months: OR = 0.26 95% CI 0.09, 0.77) were less likely to receive ORS therapy against diarrhea in HBC. The children whose family had the habit of drinking boiled water (OR = 2.77 95% CI 1.30-5.91), or whose caretakers received educational materials about childhood diseases (OR = 3.08 95% CI 1.54, 6.16), or who were living in the villages in which village clinics had the available ORS packages (OR = 3.94 95% CI 2.25, 6.90) were more likely to receive ORS therapy against diarrhea in VLC.

**Conclusion:**

There thus, ORS promoting program should give the highest priority to home care. ORS promoting strategies for low-level care could be strengthened based on children characteristics, the habit of drinking water and the situation of receiving educational material in the families and on the availability of ORS packages in village clinics in rural Western China.

## Background

Diarrhea remains a leading cause of death among infants and young children [1-4]. The major pathogenic mechanism of diarrhea mortality is dehydration, which is responsible for more than half of diarrheal deaths in developing countries [5]. Oral rehydration salts (ORS) dissolved in water to form oral rehydration solution can be absorbed in small intestine, replacing water and electrolytes lost in faeces, and is likely to produce fast recovery and fewer side effects [6,7]. So it is a safe and effective treatment administered at home or at medical centers [7]. ORS can prevent 93% of childhood diarrhea mortality [4], and the use of anti-diarrhea drugs and antibiotics, which have no clinical benefits, cannot be allowed in the treatment of acute watery diarrhea [8]. However, even up to 80% of the children with diarrhea in some areas were reported to have received no ORS but only anti-diarrheal drugs [8].

Successful reduction of diarrhea mortality in the 1970s and 1980s can be attributed largely to the scaling-up use of oral rehydration therapy and programs to educate caregivers on its appropriate use. Regional data shows that since 2000 there has been slight decline in the proportion of children receiving ORS therapy during episodes of diarrhea [9,10]. So, the simple and effective tool of child survival has fallen off the priority lists for global and national policy leaders and program managers [9]. How to sustain and achieve a high level of oral rehydration therapy continues to be a challenge [10].

Households and village clinics play an important role in the management of childhood diarrhea [1,4]. A previous report from Mongolia has showed that more than one-fifth of infant and child deaths occurred at home [11]. In rural China, village clinics located at the first tier of the rural healthcare system supply the population living in the village with many services of disease control and prevention [12]. But village doctors, who were both peasants and healthcare workers, were inclined to use medicine inappropriately [12,13]. So, it is of critical importance for childhood diarrhea to find new strategies of promoting and sustaining a high level of ORS use in home or village-level care against the diarrhea mortality. This study has assessed the utilization rate of ORS in the care of diarrhea among children under 36 months old and explored the determinants of ORS use in home-based and village-level care. Such a study can provide some insights of promoting ORS use in home-based and village-level care and reducing the deaths due to diarrhea among children under 36 months old in rural Western China.

## Methods

### Setting and study population

Supported by Chinese Ministry of Health and UNICEF, a rural primary health care survey in 45 counties of west China’s 10 provinces----Xinjiang, Inner Mongolia, Qinghai, Gansu, Ningxia, Sichuan, Chongqing, Guizhou, Jiangxi and Guangxi was conducted from June to August 2005. These 45 counties were pre-determined but the much smaller sampling units as townships and villages were sampled through a multi-stage probability-proportion-to-size sampling (PPS) method. Five townships out of each county and four villages out of each sampled township were randomly selected. In the household-sampled process, a completely random sampling method was adopted to extract sixteen households from each village. If a village had more than 16 households, 16 households were selected randomly; if a village had fewer than 16 households, all the households were determined and the rest were selected out of the neighboring villages. In every sampled household, only one child under 36 months old was selected randomly and the caretaker of the selected child was interviewed.

### Data collection

All data in the study were collected by means of pre-coded structured family and village clinic interview questionnaires. First, we had a face-to-face interview with all the caretakers involved in the survey about their families, their children and themselves after they had signed the informed consent form. All socio-demographic information in the study was included in the family questionnaire. If their children had suffered diarrheal episode in the previous two weeks (Diarrhea was defined as the passage of 3 or more loose or watery stools in the proceding 24 hours), we further interviewed them about the recent diarrheal episode in detail, including recognition of 7 dangerous symptoms of diarrhea (frequent watery stools in the proceeding one or two hours, blood in stools, repeated vomiting, high fever, extreme thirst, no desire to drink and refusal to eat), their care-seeking behaviors and ORS use in different care locations. Meanwhile, we also asked them whether they had received educational materials including the basic prevention or care knowledge of common childhood diseases which were specific in the rural primary health care program. Then, we collected the data of village clinics from village health personnel through a village clinic questionnaire about the basic information of village doctors, their medication and retail pharmacy distribution in the village. To make the collected data available, the chief of the investigation team must review every sheet of questionnaire and verify its appropriateness carefully before all questionnaires were accepted. The study was approved by the Ethics Committee of College of Medicine of Xi’an Jiaotong University.

### Variables

The outcome variables of interest included the ORS use in home-based care (HBC) and village-level care (VLC). If a child had received care at home, or in a village clinic, a township hospital or a county-level-or-above hospital in a last diarrheal episode during the previous two weeks, he/she was identified as receiving HBC, VLC, township-level care (TLC) or county-level-or-above care (CLC) respectively. HBC indicated that the caretakers gave some special care or treatment, such as increasing the frequency of feeding, giving ORS, increasing fluid intake or medical care, and so on. If a sick child was not given the above-mentioned special care at home or some care at health facilities, he/she was regarded as receiving no care (NC). In rural China, all caretakers did not have medical background. Village doctors, many of whom did not receive formal medical education, were engaged both in healthcare and farming. Therefore, we identified HBC or VLC as a low-level care. If a child with diarrhea had received ORS packets in a recent diarrheal episode in the previous two weeks and all ORS packets were administered at the corresponding care location, he/she was identified as using ORS at the care location. The caretakers’ capacity of judging the danger signs of childhood diarrhea was assessed through the number of the dangerous symptoms of diarrhea they could recognize out of the 7 ones. The Demographic and Health Survey (DHS) wealth index generated with the five variables (type of vehicle, water supply, income resource, texture of pot and type of television) was used to assess the socioeconomic status of the families [14,15]. According to the tertiles of the DHS wealth index, the economic statuses of the families fell into three: poor, medium and rich [14,15].

### Data analysis

The data from the qualified questionnaires was entered in Epidata 3.1 by double entry. SPSS version 17 (SPSS Inc, Chicago, IL, USA) was employed to make the statistical analysis. The chi-square test was adopted to compare the proportions. Generalized estimated equation (GEE) logistic regression models were used to predict the determinants of ORS use in HBC and VLC respectively while controlling for the possible correlation of ORS use in the same village. The level of the significance of analysis was set at 0.05.

## Results

### Sample characteristics and village-level information

The study investigated 14112 households and 894 villages totally. In these surveyed households, 1040 children had suffered at least one of diarrheal episodes in the previous two weeks. Table 1 shows sample characteristics and village-level information by HBC and VLC. Extra analysis of care-seeking behavior (results not displayed) showed that of the children with diarrhea, about 9% received NC, slightly less than 15% received HBC, approximate 40% sought VLC, 27.3% were sent to township hospitals for diarrhea and 9.52% sought the care in county-level or above medical sectors.

**Table 1:** Sample characteristics and village-level information by HBC and VLC

**Information**	**All n (%)**	**HBC n (%)**	**VLC n (%)**
**Household-level information**	1040(100.00)	153(14.71)	413(39.71)
Number of family members (<4)	228(21.92)	46(30.07)	84(20.34)
Number of pre-school-aged children (>1)	456(43.85)	63(41.18)	183(44.31)
Drinking boiled water usually	839(80.67)	119(77.78)	343(83.05)
Economic status of the family			
Rich	275(26.44)	36(23.53)	134(32.45)
Medium	209(20.10)	31(20.26)	79(19.13)
Poor	556(53.46)	86(56.21)	200(48.43)
Mother care	873(83.94)	136(88.89)	325(78.69)
Han ethnicity	551(52.98)	49(32.03)	250(60.53)
Age of mothers (25–34 year)	562(54.04)	74(48.37)	249(60.29)
Maternal education (0–9 years)	964(92.69)	145(94.77)	400(96.85)
Receiving educational materials about childhood diseases	823(79.13)	117(76.47)	330(79.90)
The number of danger signs recognized (mean, SD)	2.92,1.85	2.46(1.26)	3.08(1.96)
Age of diarrhea children			
0-12 months	509(48.94)	59(38.56)	191(46.25)
13-24 months	360(34.62)	56(36.60)	147(35.59)
25-36 months	171(16.44)	38(24.84)	75(18.16)
Boy	615(59.13)	86(56.21)	247(59.81)
Breastfeeding when surveyed	534(51.26)	75(49.02)	197(47.70)
Oral vitamin A in the previous year	583(56.06)	89(58.17)	243(58.84)
^b^**Village-level information** (n)			
Number of retail pharmacies (≥1)	63(11.93)	7(6.49)	45(15.68)
Number of village doctors (one doctor)	321(60.80)	77(71.30)	258(89.90)
Age of village doctors (≥35 years)	360(68.18)	72(66.67)	182(66.90)
Education of village doctors(Technical school and above)	266(50.38)	43(39.81)	162(56.45)
Practice period of village doctors(≥10 years)	297(56.25)	65(63.11)	170(59.23)
Available ORS in village clinic	208(39.39)	22(20.37)	134(46.69)

^*a*^HBC, home-based care, VLC, village-level care.

^*b*^The information of nine villages was missing.

Additionally, all children with diarrhea were living in 537 villages of 204 townships. Of these villages, most had no retail pharmacies and about 60% had only one village doctor. Only two-fifths of village clinics had some ORS available (Table 1).

### Use of ORS

**Figure 1:**
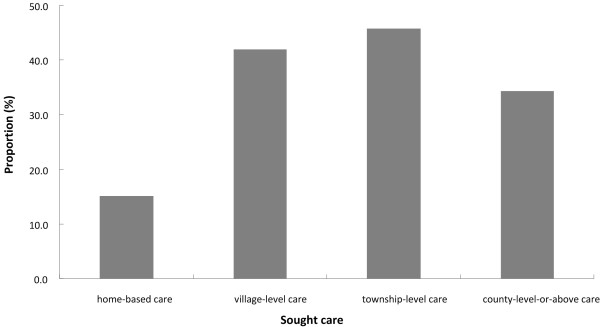
ORS use in the sought care of diarrhea among children under 36 months old in rural Western China.

**Figure 2:**
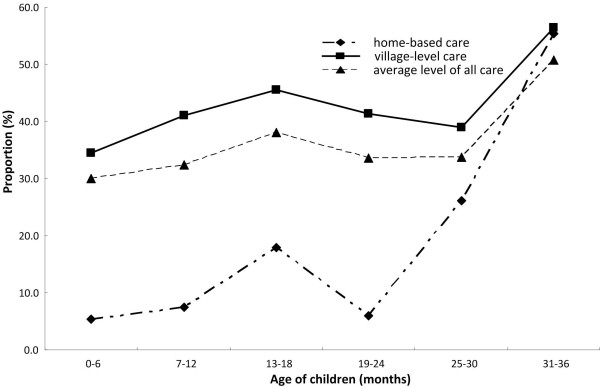
ORS use for diarrhea in the 6 age-groups by home-base care, village-level care and average level of all care among children under 36 months old in rural Western China.

Table 2 shows ORS use for diarrhea among children under 36 months old in 10 provinces of rural Western China. The overall utilization rate of ORS was 34.62% (95% CI: 31.72, 37.51). In the four kinds of care, the utilization rate of ORS in TLC was the highest (45.77%), that in HBC the lowest (only 15.03%), and that in VLC and in CLC 41.89% and 34.34% respectively. Figure 1 shows the utilization rate of ORS in different care. There was a significant difference in ORS use among the 4 care groups (*x*^2^ = 32.47, p < 0.001). By six pairwise comparisons, the utilization rate of ORS in HBC was significantly lower than that in VLC, TLC or CLC. (HBC vs VLC: p < 0.001; HBC vs TLC: p < 0.001; HBC vs CLC: p < 0.001). Figure 2 shows the utilization rate of ORS in HBC, VLC and average level at all care in the 6 age-groups (0-6 months, 7-12 months, 13-18 months, 19-24 months, 25-30 months and 31-36 months). In the 6 age-groups, we observed a significant difference in ORS use (*x*^2^ = 14.31, p = 0.026) when controlling for breastfeeding status. Among 15 pairwise comparisons, four pair was significantly different (6 vs 36 months: p = 0.004; 12 vs 36 months: p = 0.009; 24 vs 36 months: p = 0.017; 30 vs 36 months: p = 0.032).

**Table 2:** ORS use for diarrhea among children under 36 months old in 10 provinces of rural Western China

**Province**	**Number of households surveyed (n)**	**The number of children with diarrhea in previous two weeks (%)**	**ORS use**	
			**n**	**%**
Gansu	634	27 (4.26)	6	22.22
Guangxi	1586	58 (3.66)	19	32.76
Guizhou	1265	116 (9.17)	43	37.93
Jiangxi	1567	74 (4.72)	42	56.76
Inner Mongolia	1217	65 (5.34)	5	7.69
Ningxia	1264	79 (6.25)	29	37.18
Qinghai	1589	119 (7.49)	32	26.67
Sichuan	1577	158 (10.02)	94	59.49
Xinjiang	2168	315 (14.53)	75	23.81
Chongqing	1245	29 (2.33)	14	48.28
All	14112	1040 (7.37)	360	34.62

ORS = oral rehydration salts.

### Determinants of ORS use in low-level care

Table 3 shows the predictors of ORS use in HBC and VLC among children with diarrhea under 36 months old. GEE model analysis of ORS use in HBC demonstrated that the caretakers with more than one child seemed less likely to use ORS in recent diarrheal episode and that the younger children were less likely to use ORS in HBC. GEE model analysis of ORS use in VLC demonstrated that the families’ habit of drinking boiled water, caretakers’ receiving educational materials about childhood diseases and ORS available in village clinics seemed to increase the likelihood of ORS use.

**Table 3:** Predictors of ORS use of diarrhea children below 36 months old in the low-level care in rural Western China

**Variables**	**Univariate**		^**d**^**Multivariate**	
	**OR**	**95% CI**	**OR**	**95% CI**
^*a,c*^ORS use in HBC				
Number of pre-school-age children				
>1	0.46	0.19,1.10	0.29	0.10,0.86
1	1		1	
Age of children				
0-12 months	0.17	0.05,0.59	0.10	0.02,0.41
13-24 months	0.43	0.16,1.15	0.26	0.09,0.77
25-36 months	1		1	
^*b*^ORS use in VLC				
Drinking water				
Boiled water often	2.82	1.57,5.05	2.77	1.30,5.91
No or boiled water occasionally	1		1	
Receiving educational materials about childhood diseases				
Receiving	3.52	2.03,6.11	3.08	1.54,6.16
Non-receiving	1		1	
Available ORS in village clinics				
Yes	3.96	2.54,6.19	3.94	2.25,6.90
No	1		1	

^*a*^Fourteen household-level variables were entered together into GEE logistic model.

^*b*^Fourteen household-level variables and six village-level variables were together entered into GEE logistic regression model. ^*c*^HBC = home-based care; VLC = village-level care; ORS = oral rehydration salt; OR=odds ratio; CI = credible interval.

^d^Only predictors at 5% of multivariate analysis model were listed.

## Discussion

Oral rehydration therapy is the cornerstone of fluid replacement and national programs to promote ORS have been strongly supported by WHO, UNICEF and USAID in the treatment of diarrhea [1,16]. Our study found that the overall therapy rate of ORS in all care of diarrhea among children under 36 months old is 34.62%. In the 4 care groups, the utilization proportion of ORS in HBC was only 15.03%, significantly lower than that in VLC, TLC or CLC. It was clear that these caretakers did not use ORS against childhood diarrhea in HBC more often. In our study, overwhelming majority of the mothers of these children completed only a primary education. It is possible that this may contribute to less education about when to appropriately use ORS. The previous study also showed that the devalued status of ORS in the eyes of caretakers had become a major problem [17]. It should be acknowledged that these children with diarrhea in HBC usually had symptoms not as severe as those who were taken to a formal healthcare facility. These may lead to the low utilization of ORS in HBC. So programs of promoting ORS use should give a significant priority to the households. Meanwhile, communication strategies are needed to ensure that families understand and accept ORS as a key treatment component in HBC [1]. Also, there should be an urgent need for caregivers to be educated to use ORS packets at home as early as possible when a diarrheal symptom appeared in their children. GEE model analysis of ORS use in HBC found that the caretakers who cared for more than one child seemed less likely to use ORS in recent diarrheal episode. In the multi-child families in rural China, it is common for the caretakers to delegate some care burdens to their older children. However, other children in the household may not have the appropriate knowledge or skills to care for a younger sibling with diarrhea. The demands of caring for multiple children negatively impacted caretakers’ ability to provide appropriate and timely diarrheal treatment for the ill child. This may contribute to low use of ORS packets for sick child in families with multiple children. Our study also found that the younger children were less likely to use ORS in HBC. Due to the fact that younger children were more likely to be breastfed or fed with more liquid food, caretakers would not like to think it necessary to use ORS frequently in their children with diarrhea in such a feeding period [18]. In addition, a similar study of ORS therapy in rural Bangladesh demonstrated that the mothers generally had the perception that infants should not drink any fluids other than breast milk before this age, and the infants were introduced to water and other clear fluids after this age [19]. In rural China, such a perception among the caretakers in home care may also be a factor, explaining why children less than 12 months old were offered ORS less frequently in HBC than those aged 25–36 months. So health communications should specifically inform caregivers that ORS can be used in sick children who are currently being breastfed.

The utilization rate of ORS in VLC (41.89%) was only lower than that in TLC (45.77%). Recent study of prescriptions of village doctors in these areas has shown that in the village clinics more than one-third of the doctors had no full-time medical education and village doctors were inclined to adopt inappropriate drug utilizations in the treatment of diarrhea [12,13]. More educational or training projects about appropriate and early ORS use by the government should be carried out in village-level medical sectors urgently. GEE model analysis of ORS use in VLC showed that ORS use was positively associated with the habit of drinking boiled water often in the families. Families that drank boiled water often at home may have more faith in ORS as a treatment or consider it standard treatment of childhood diarrhea. When they took their children to village clinics, they also agreed that village doctors could use ORS for their children with diarrhea. Our study also showed that receiving educational materials about childhood diseases was more likely to increase the likelihood of ORS use in VLC. The prescription of ORS in village clinics seemed to meet with the profile of educational materials about the treatment of childhood diarrhea and thus made the caretakers more likely to believe in the decision of village doctors to use ORS. Our study also found that ORS available in village clinics was more likely to increase the use of ORS, as found by a study of ORS use at home [20]. Thus, when ORS packets were not available in village clinics, self-made ORS based on the WHO formulation could be used to treat children with diarrhea.

### Limitation

Some limitations of our study should be acknowledged. As it had not been designed to collect all the information about ORS use, some important information such as the dose, type and frequency of ORS therapy and so on was not available, which could affect our results. Moreover, all data were collected by self-report, which might involve some recall biases. Besides, some other confounding factors which might potentially affect the incidence of childhood diarrhea such as the severity of childhood diarrhea, might also affect ORS use. In addition, use of ORS in HBC or care of clinic or hospital will likely depend on the perceived severity of the diarrhea. But this survey did not collect the information about severity of childhood diarrhea. This also may affect our study results potentially.

## Conclusion

In conclusion, the utility rate of ORS in HBC was the lowest in all sought care. So, ORS promoting program should give the highest priority to home care. The number of pre-school-aged children in a family and the children’s ages affected ORS use in HBC. The families’ habit of drinking boiled water, educational materials about childhood diseases and the availability of ORS in village clinics affected ORS use in VLC. ORS promoting strategies for low-level care could be strengthened based on children characteristics, the habit of drinking water and the situation of receiving educational material in the families and on the availability of ORS packages in village clinics in rural Western China.

## Competing interest

All authors declare no conflict of interest.

## Authors’ contributions

WG designed the study, conducted the data analysis and prepared the manuscript; HY directed the study and the writing; DW assisted with the data analysis and reviewed the manuscript; SD directed and reviewed the manuscript. All authors have read and approved the final manuscript.

## Pre-publication history

The pre-publication history for this paper can be accessed here:

http://www.biomedcentral.com/1471-2458/13/238/prepub
